# Effect of intervention based on the 5A self-management model on the improvement of behaviors related to women's reproductive health after legal abortion

**DOI:** 10.1038/s41598-023-38521-y

**Published:** 2023-07-13

**Authors:** Zahra Mirian, Mahshid AbdiShahshahani, Mahnaz Noroozi, Firozeh Mostafavi, Marjan Beigi

**Affiliations:** 1 Student Research Center, Faculty of Nursing and Midwifery, Isfahan University of Medical Sciences, Isfahan, Iran; 2 Department of Midwifery and Reproductive Health, Faculty of Nursing and Midwifery, Isfahan University of Medical Sciences, Isfahan, Iran; 3 Department of Health Education and Health Promotion, Faculty of Health, Isfahan University of Medical Sciences, Isfahan, Iran

**Keywords:** Health care, Medical research

## Abstract

Legal abortion has adopted many beneficial measures to prevent the birth of fetuses with congenital anomalies and maintain the health of high-risk mothers. However, abortion has its own complications and, thus, most mothers need care in this regard. The aim of this study was to determine the effect of self-management model on behaviors related to the reproductive health of mothers after legal abortion. This clinical trial study was conducted on 72 women who had legal abortion with the permission from Isfahan forensic medicine. The intervention was conducted using the 5A self-management model. The control group received the usual health system cares. Data collection tool was a researcher-made questionnaire of behaviors related to reproductive health, which was completed before and two months after the intervention by the two groups. The total mean score of the behavior (P < 0.001) and its dimensions (Prevention of post-abortion complications with P = 0.01, Prevention of sexually transmitted infections, satisfactory sexual function and family planning with P < 0.001) in the intervention group two months after the outset of the intervention was significantly higher than before the intervention. The total mean score of the behavior (P < 0.001) and its dimensions (Prevention of post-abortion complications with P = 0.002, Prevention of sexually transmitted infections, satisfactory sexual function and family planning with P < 0.001) two months after the outset of the intervention in the intervention group was significantly higher than the control group. Based on the results, this educational model can be emphasized to promote reproductive health behaviors of women after abortion.

Clinical trial protocol link: https://www.irct.ir/user/trial/55184/view.

## Introduction

In some countries, especially Muslim countries, legal abortion is defined as the termination of pregnancy because of the threats it may pose to the mother's life as well as fetal congenital anomalies. However, in some other countries, in addition to the mentioned reasons, the mother's request is also important^1^. Although legal abortion has adopted useful measures to prevent the birth of fetuses with congenital anomalies and terminate pregnancy to save mother's health, it should be noted that abortion has its own complications^2^. Abortion-related complications are the most important but preventable causes of maternal mortality, which account for 8–9% of maternal deaths worldwide, as 42–63 women out of every 100,000 die because of abortion^3, 4^. In order to prevent abortion-related maternal mortalities, high-quality post-abortion care (PAC) should be provided at all levels of the healthcare system, including rural and urban health centers^5, 6^. To this end, post-abortion care (PAC) program has been designed in many countries^7^. The main objective of these cares is to involve society and health service providers in providing appropriate services and timely care to prevent post-abortion complications, offer family planning counseling, identify sexual and reproductive health (SRH) needs and respond to them^8^. According to the guidelines of the World Health Organization (WHO) and the International Federation of Gynecology and Obstetrics (FIGO), women need to avoid pregnancy for at least 4 to 6 months after abortion so that they can have a safe pregnancy^9^. Therefore, these women need to be provided with family planning counseling immediately after abortion and within 48 h^10^. Early consideration of post-abortion complications, such as bleeding and infection, and their treatment are crucially important as they may threaten the life of the mother^11^.

In the present era, when healthy fertility and childbearing is a primary goal of policymaking in societies, it is essential to prioritize and support women's reproductive health. In this context, providing women with high-quality post-abortion care that includes education, counseling and support is crucial for improving their reproductive health outcomes^12, 13^. As such, the use of various educational theories and models to change health behavior can have a double impact^14, 15^. One effective method for promoting individual health based on their abilities is the 5A self-management model^16^, also known as the behavior change model that is used for promoting positive health behaviors^17, 18^. This self-management model consists of 5 steps: the first step is assessment of the identified problems of each person; the second step is advice which is provided based on the results of the assessment and the clients are informed about their problems and the risks caused by those problems; in the third step, agreement, an action plan is agreed upon which includes a set of realistic goals; in the fourth step or assistance, some clients may need specialized education or counseling, which is provided by introducing them to counselors, as well as through providing them with in-person oral education or brochures; and the fifth step is arrangement according to which the status of behavioral goals and practical plans, counseling and encouragement to continue the intervention are arranged and checked through phone calls and visits to the clinic or home^16, 19^.

As mentioned earlier, to change the behavior of individuals, their awareness must be increased through education, and the best health behaviors follow appropriate educational planning^20, 21^. There are few studies regarding the use of educational models of behavior change for maternal health services and, hence, there is an obvious need for conducting more studies in the area of post-abortion services. The use of health behaviors by mothers after doing abortion is a requirement of the health system because the maternal health index is considered a development index, and there are global efforts to improve it^4^. The present study, thus, was designed to determine the effect of the intervention based on A5 self-management model on the promotion of reproductive health-related behaviors of women after legal abortion.

## Methods

This clinical trial study with a control group was approved by the Ethical Committee of Isfahan University of Medical Sciences (IR.MUI.RESEARCH.REC.1399.819/ date of, 09.11.2020) and was conducted in 18 hospitals (educational, general, private, charity and institution-affiliated hospitals) in Isfahan city. It was also registered in clinical trial databases (IRCT20210315050716N1/ date of registration: 28.05.2021).

### Participants

The research population consisted of all women who, based on the license issued by the Isfahan Forensic Medicine Center, underwent legal abortion in 18 hospitals (educational, general, private, charity, and institution-affiliated) in Isfahan during the data collection period. The researcher was informed about the presence of research samples in the hospital by the head of the hospital.

The inclusion criteria were women of reproductive age, who had undergone legal abortion based on the legal abortion license issued by Isfahan Forensic Medicine Center, were willing to participate in the study, had access to a smartphone and high-speed Internet, did not participate in other clinical studies at the same time, and were at least semiliterate. Exclusion criteria included the incidence of mental and physical problems during the research to the extent that made the participation impossible, the incidence of sudden and serious stress before completing the questionnaire of the second stage (e.g., death of relatives, etc.), and not attending the educational sessions.

### Tools

The data collection tool was a two-part researcher-made questionnaire, the first part of which included demographic characteristics and the second part included the questions related to behaviors of reproductive health. Demographic characteristics questionnaire included age, age of spouses, number of children, education level and marriage duration. Questionnaire for the evaluation of behaviors related to reproductive health of the participants included 26 items in 4 areas of prevention and management of post-abortion complications (5 items), prevention of sexually transmitted infections (8 items), satisfactory sexual function (7 items) and family planning (6 items). The questions of the questionnaire were scored on a 5-point Likert scale (1 = never, 2 = rarely, 3 = sometimes, 4 = often, 5 = always). The total score of reproductive health-related behaviors ranged from 26 to 130, that the scores obtained in this study were expressed as percentage. The total mean score of the behavior and its dimensions in the intervention and control groups was calculated before and after the implementation of the 5A self-management program.

The content validity was tested by 12 experts. The CVI and CVR were calculated to be 0.90 and 0.94, respectively. Moreover, the face validity and reliability of this questionnaire were examined in a pilot study on 40 women. According to the recommendations of the experts, minor revisions were made to increase the transparency and comprehensibility of the statements. The reliability of this questionnaire was also measured by internal consistency (Cronbach's alpha = 0.868).

### Sampling

The samples were selected using the convenience sampling method and then random number table were used for random assignment of the two intervention and control groups. Considering the equation N = [(Z1 + Z2)^2^(2S^2^)]/d^2^ (d = 0.75, Z1 = 1.96, Z2 = 0.84), the number of samples were calculated to be 32 subjects in each group; however, taking into account the probable drop of 10% in the samples, 36 subjects were considered for each group and the total number of the samples was 72.

**Figure 1:**
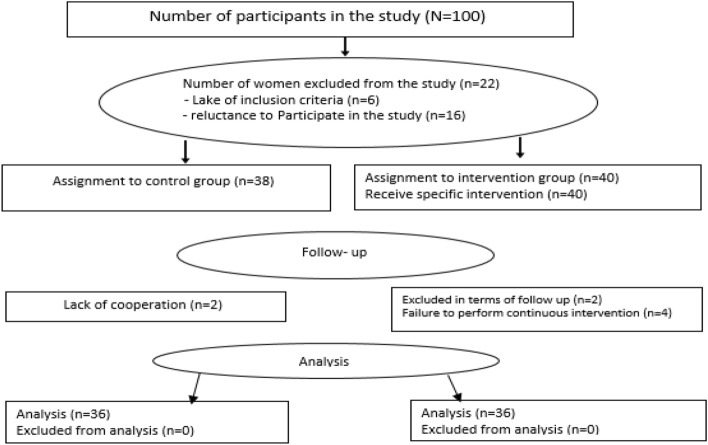
The CONSORT flow diagram of the study.

Out of 38 subjects invited for the control group and 40 subjects invited for the intervention group, 36 subjects in each group accepted to participate in the study (Fig. 1). The age range of the subjects was 22–43 and 20–49 in the intervention and control groups respectively.

### Intervention

This clinical trial study was conducted with two intervention and control groups to determine the effect of the intervention based on the 5A self-management model on reproductive health-related behaviors of women. The researcher went to medical centers after the research was approved by the ethics committee of the university. هAll ethical considerations were taken into account based on the Declaration of Helsinki.

After reviewing the records of women who underwent legal abortion, eligible women were selected based on the inclusion criteria. The samples were then randomly assigned to either the intervention and control group using a random number table. Prior to the intervention, informed consent was obtained from all participants. Both intervention and control groups completed a demographic characteristics questionnaire and the researcher-made questionnaire to measure reproductive health-related behaviors. The control group received routine cares based on hospital educations. However, to comply with ethical considerations, the educational file was provided to them after the completion of the intervention.

The intervention group was subjected to an intervention based on the 5A self-management model, implemented in five stages.

1. *Assessment stage* In this stage, the research samples were evaluated based on their fertility history, level of awareness of and attitude towards acquiring and promoting reproductive health in terms of sexual performance, compliance with reproductive health practices and belief in family planning, and the desired information was obtained from them. This stage involved individual, face-to-face interview as well as a researcher-made questionnaire on reproductive health, which was carefully reviewed by the researcher after completion by the research participants.
2. *Advice stage* After conducting individual and face-to-face interviews, we informed the research participants about the importance of reproductive health behaviors after abortion. Specifically, we explained the risks of not performing these behaviors and the benefits of engaging in the desired behaviors. We provided person-to-person guidance to each participant, taking into account their cultural and social background. The guidance was delivered through face-to-face interaction and in simple language.
3. *Agreement stage* In this stage, necessary and appropriate educational goals and needs were designed. After that, an agreement was reached on the determined needs according to the interests of the research samples as well as their willingness and ability to improve their behavior. In fact, at this stage, educational interventions were designed for the most important educational needs.
4. *Assistance* This fourth stage of the research occurred one week after the third one. The participants received the necessary education about reproductive health and prevention of sexually transmitted infections, satisfactory sexual function, and family planning through two 2-h educational sessions (one session per week). The educational document, which included the above information, was provided to the participants. It should be noted that given the Covid-19 pandemic, in-person classes were not possible, and the classes were held online with the possibility of communicating with the clients through audio and video calls to facilitate effective interactions between the participants and the researcher. If participants required professional counseling, they were referred to an expert.
5. *Arrangement* The function of the participants was monitored in this stage to ensure the practical plans were implemented as intended. Daily follow-up was conducted for 2 weeks, followed by twice-weekly phone calls. Moreover, the progress and success of the participants in following reproductive health-related behaviors and their need for referral were assessed. If changes to the goals or practical plan were necessary, they were agreed upon. Additionally, if the participants required more educations, it was provided during the follow-up period. The researcher provided the intervention group with a telephone number so that they could call for problem-solving at any time of day.

**Table:** 

Self-management model with 5A	
Assessment	The level of awareness of the participants and their attitude towards reproductive health behaviors after abortion was determined
Advice	The risks of not performing the reproductive health-related behaviors as well as the benefits of engaging in the desired behaviors were explained to research samples
Agreement	Educational goals and needs were determined with the agreement of the research samples
Assistance	The research subjects were given the necessary education about reproductive health and prevention of sexually transmitted infections, satisfactory sexual function and family planning in two 2-h educational sessions (one session per week)
Arrangement	The function of the patients was followed up in this stage. In fact, in order to ensure the implementation of the practical plans by the research samples, daily follow-up was done for 2 weeks and after that twice a week through telephone calls

The trainings were scientific and explained in simple words. The participants had access to educational materials through Sky room. Sky room link was provided to the participants by the researcher. The women of the control group received only the routine hospital cares while the interventional materials were provided to them after the completion of the intervention to comply with ethical considerations.

Two month after the start of the intervention, both the intervention and control groups completed the questionnaire for evaluating the behaviors related to reproductive health.

### Data analysis

The data collected were analyzed using web-based SPSS software version 19. Moreover, the independent t-test, paired t-test and Mann–Whitney statistical test were used for data analysis. A significance level of less that 0.05 was considered in all stages of the data analysis.

### Ethics approval and consent to participate

All respondents were assured that their information would remain confidential and anonymous. Written consent was obtained from the participants to enter the study. Ethical approval for this study has been obtained by the ethics committee affiliated with Isfahan University of Medical Sciences, Isfahan, Iran (IR.MUI.RESEARCH.REC.1399.819).

## Results

The intervention based on the 5A self-management model was implemented continuously in all participants and none of them were excluded from the study. Based on the results of the statistical tests and random allocation of the participants, no significant difference was observed in the demographic and fertility variables of the them such as age of mothers, age of spouses, number of children, marriage duration and education level between the two groups (p > 0.05) (Tables 1, 2).

**Table 1:** Demographic and reproductive information of the intervention and control groups.

Dimensions	Group	M(S)	Inter group change		
			T	df	P
Mother's age (years)	Intervention group	34.92 ± 5.55	0.91	70	0.37
	Control group	33.47 ± 7.76			
Age of spouses (years)	Intervention group	38.08 ± 5.17	0.25	70	0.81
	Control group	37.72 ± 7.09			
Number of children	Intervention group	1.19 ± 0.98	0.39	70	0.70
	Control group	1.28 ± 0.85			
Length of marriage (years)	Intervention group	11.41 ± 6.45	0.19	70	0.85
	Control group	11.11 ± 6.75

**Table 2:** Educational information of the intervention and control groups.

	Dimensions	Group	N (%)	Mann–Whitney test	
				Z	P
Educations level of education	High school	Intervention group	8 (22.20%)	0.88	0.15
		Control group	9 (25%)		
	Diploma	Intervention group	14 (38.90%)		
		Control group	12 (33.30%)		
	Masters	Intervention group	11 (30.60%)		
		Control group	14 (38.90%)		
	Masters and above	Intervention group	3 (8.30%)		
		Control group	1 (2.80%)		

According to the independent t-test, the total mean score of the behavior and its dimensions before the intervention did not differ significantly between the two groups (P > 0.05) (Table 3). Based on the paired t-test, the total mean score of the behavior and its dimensions in the intervention group two months after the intervention was significantly higher than before the intervention (P < 0.05) (Table 4).

**Table 3:** Comparison of the total mean score of behavior and its dimensions before and two months after the outset of the intervention between the two groups.

Dimensions	Group	Before the intervention	After 2 months	After 2 months			Before the intervention		
		M(S)	M(S)	T	df	P	T	df	P
Total	Intervention group	107.42 (15.08)	126.72 (3.01)	0.66	70	0.51	8.29	70	< 0.001
	Control group	104.86 (17.75)	102.92 (16.96)						
Prevention and management of post-abortion complications	Intervention group	23.89 ± 2.34	24.89 ± 0.52	0.09	70	0.93	3.30	70	0.002
	Control group	23.94 ± 2.76	23.69 ± 2.11						
Prevention of sexually transmitted infections	Intervention group	33.25 ± 7.25	39.11 ± 1.53	1.36	70	0.18	7.95	70	< 0.001
	Control group	31 ± 6.73	30.42 ± 6.38						
Satisfactory sexual function	Intervention group	27.86 ± 6.68	33.31 ± 1.98	0.03	70	0.97	6.17	70	< 0.001
	Control group	27.80 ± 7.74	26.64 ± 6.17						
Family planning	Intervention group	22.42 ± 4.99	29.42 ± 0.87	0.23	70	0.82	6.83	70	< 0.001
	Control group	22.1 ± 6.34	22.17 ± 6.31

**Table 4:** Comparison of the total mean score of behavior and its dimensions in the intervention group and the control group between the two times.

Dimensions	Group	Before the intervention	After the intervention (after 2 months)	Inter group change		
		M(S)	M(S)	T	df	P
Total	Intervention group	107.42 (15.08)	126.72 (31.01)	7.90	35	< 0.001
	Control group	104.86 (17.75)	102.92 (16.96)	1.30	35	0.20
Prevention and management of post-abortion complications	Intervention group	23.89 ± 2.34	24.89 ± 0.52	2.60	35	0.01
	Control group	23.94 ± 2.76	23.69 ± 2.11	0.72	35	048
Prevention of sexually transmitted infections	Intervention group	33.25 ± 7.25	39.11 ± 1.53	5	35	< 0.001
	Control group	31 ± 6.73	30.42 ± 6.38	1.07	35	0.29
Satisfactory sexual function	Intervention group	27.86 ± 6.68	33.31 ± 1.98	5.59	35	< 0.001
	Control group	27.80 ± 7.74	26.64 ± 6.17	1.35	35	0.18
Family planning	Intervention group	22.42 ± 4.99	29.42 ± 0.87	8.85	35	< 0.001
	Control group	22.11 ± 6.34	22.17 ± 6.31	0.25	35	0.80

The paired t-test indicated that the total mean score of the behavior and its dimensions in the control group was not significantly different between the two times (P > 0.05) (Table 4). Finally, the independent t-test revealed that the total mean score of behavior and its dimensions in the intervention group two months after the start of the intervention was significantly higher than the control group (P < 0.05) (Table 3).

## Discussion

The present study aimed to determine the effectiveness of an intervention based on the 5A self-management model on improving of reproductive health-related behaviors in women after legal abortion. The study's initial findings indicated that there was no significant difference was observed between the two groups in terms of the demographic and reproductive information, which suggests that the random allocation method was successful. The matching of demographic and reproductive characteristics has been addressed in other clinical trials based on the 5A model^22–24^.

Based on the results, the total mean score of behavior and its dimensions in the intervention group two months after the intervention was significantly higher than before the intervention. However, there was no significant difference in the total mean score of behavior and its dimensions in the control group between the two times. These findings suggest that the self-management training program had a significant impact on improving reproductive health-related behaviors of the intervention group, that is, the training program could modify reproductive health-related behaviors. Abortion self-management has been investigated in several previous studies, which have also highlighted the importance of self-management interventions in promoting reproductive health.

Assessing 99 studies on self-management of abortion and post-abortion care, Moseson et al. reported that the self-management of abortion care and post-abortion complications was effective in improving reproductive health outcomes in volunteer women^25^. In their study entitled "health care providers' views on self-management of abortion", Baldwin et al. argued that women have the right to manage their own abortion and control its complications and the creating self-efficacy among these women along with supervision of supervisors can lead to healthy fertility outcomes^26^. In their study entitled "the effectiveness of self-management process of abortion and its care in Nigeria and Argentina", Moseson et al. found that while self-management of abortion was effective, it was more effective when accompanied by support-advisory groups^27^. Fuentes et al. emphasized the importance of empowering women to perform self-induced abortion safely through proper training and clinical advisor ^28^. Egwuatu et al. indicated in their study titled "the effectiveness of self-management of abortion during the Covid-19 crisis", that all abortion procedures (during abortion and aftercare) were successful for the female volunteers who had followed abortion training. They found that self-management of abortion during the Covid-19 crisis was effective, but recommended the need for an emergency hotline to address possible complications during post-abortion care^29^. Wang et al. in China highlighted the value of self-management training for post-abortion care, particularly during crises like the Covid-19 pandemic when quarantine is required to be maintained. They also emphasized the use of contraceptive methods after abortion to prevent the recurrence of induced abortions^30^. The present study is similar to the aforementioned studies in that it highlights the effectiveness of the self-management education in improving reproductive health outcomes. However, the present study differs in that it focuses on women who underwent abortion in a hospital setting and then received post-abortion self-care after being discharged to prevent the possible complications and improve the behaviors related to reproductive health.

In Iran, access to abortion services is restricted and is only performed in cases of medical indication and in hospitals. Nevertheless, special attention is given to post-abortion care in Iran to maintain the reproductive health of women, which is crucial for the country's health system. As another difference, while the 5A self-management model was used in the present study, no specific educational model was used in the aforementioned studies, yet all studies reported the success of self-management education in improving reproductive health outcomes. Not only these studies highlight the valuable impact of education on reproductive system, they also emphasize the potential of women to achieve empowerment and self-efficacy in maintaining their reproductive health. Therefore, it is important to emphasize self-management of abortion through education, particularly through the use of educational models.

One of the limitations of the present study is that the research was implemented in Isfahan city. For more generalizability, more studies can be conducted in Isfahan province.

## Conclusion

The use of self-management educations in various areas can contribute to improved learning outcomes for individuals. According to the present study, the 5A self-management model is a powerful self-care method that emphasizes individual cooperation in promoting positive reproductive health outcomes.

## Data Availability

All necessary data related to the research presented within the article. However, upon request, corresponding author can provide any additional data if needed.

## Abbreviations

5A: Assess, advise, agree, assist, arrange
PAC: Post abortion care
